# Texture-based brain networks for characterization of healthy subjects from MRI

**DOI:** 10.1038/s41598-023-43544-6

**Published:** 2023-09-29

**Authors:** Rafael Vinícius da Silveira, Li Min Li, Gabriela Castellano

**Affiliations:** 1 Department of Cosmic Rays and Chronology, Gleb Wataghin Physics Institute, University of Campinas – UNICAMP, R. Sérgio Buarque de Holanda, 777, Cidade Universitária Zeferino Vaz, Campinas, SP 13083-859 Brazil; 2 Department of Neurology, School of Medical Sciences, University of Campinas – UNICAMP, R. Tessália Vieira de Camargo, 126, Cidade Universitária Zeferino Vaz, Campinas, SP 13083-887 Brazil; 3 Brazilian Institute of Neuroscience and Neurotechnology - BRAINN, Campinas, SP 13083-887 Brazil

**Keywords:** Network models, Image processing, Network topology

## Abstract

Brain networks have been widely used to study the relationships between brain regions based on their dynamics using, e.g. fMRI or EEG, and to characterize their real physical connections using DTI. However, few studies have investigated brain networks derived from structural properties; and those have been based on cortical thickness or gray matter volume. The main objective of this work was to investigate the feasibility of obtaining useful information from brain networks derived from structural MRI, using texture features. We also wanted to verify if texture brain networks had any relation with established functional networks. T1-MR images were segmented using AAL and texture parameters from the gray-level co-occurrence matrix were computed for each region, for 760 subjects. Individual texture networks were used to evaluate the structural connections between regions of well-established functional networks; assess possible gender differences; investigate the dependence of texture network measures with age; and single out brain regions with different texture-network characteristics. Although around 70% of texture connections between regions belonging to the default mode, attention, and visual network were greater than the mean connection value, this effect was small (only between 7 and 15% of these connections were larger than one standard deviation), implying that texture-based morphology does not seem to subside function. This differs from cortical thickness-based morphology, which has been shown to relate to functional networks. Seventy-five out of 86 evaluated regions showed significant (ANCOVA, *p* < 0.05) differences between genders. Forty-four out of 86 regions showed significant (ANCOVA, *p* < 0.05) dependence with age; however, the R^2^ indicates that this is not a linear relation. Thalamus and putamen showed a very unique texture-wise structure compared to other analyzed regions. Texture networks were able to provide useful information regarding gender and age-related differences, as well as for singling out specific brain regions. We did not find a morphological texture-based subsidy for the evaluated functional brain networks. In the future, this approach will be extended to neurological patients to investigate the possibility of extracting biomarkers to help monitor disease evolution or treatment effectiveness.

## Introduction

The use of functional brain networks to characterize cognitive processes in both healthy subjects and patients with various neurological diseases has been widely spread (for a review, see e.g.^1^). Data obtained from techniques such as electroencephalography (EEG)^2–7^, magnetoencephalography^8,9^, near-infrared spectroscopy^10–13^, positron emission tomography (PET)^14,15^ and mainly, functional magnetic resonance imaging (fMRI)^16,17^, have been used for this purpose. Indeed, it has been argued that, since most brain functions depend on several areas, the network approach gives a more complete picture of brain processes and function, compared to looking at isolated brain regions (see, e.g.,^18^).

One of the first functional networks found was the default mode network (DMN); discovered by Raichle’s group using PET scans^19,20^ and later confirmed by Greicius and colleagues using resting-state fMRI^21^. It is composed of a set of regions that are thought to be involved in mind wandering or unfocused mental tasks; these regions are only active during passive rest and are ‘turned off’ when an individual engages in an externally goal-directed task. This network has been ‘measured’ in all sorts of populations, from children to various types of neurological patients, and therefore has been extensively studied. Another important resting-state functional network is the sensory-motor network (SMN), which contains the postcentral and precentral gyrus, regions associated with the somatosensory and motor cortexes, respectively. It has been shown to be active during motor tasks, such as finger tapping^22^. Other resting-state functional networks reported are the dorsal attention network (DAN), involved in the managing of the gaze-centered attention priorities^23^; the visual network, responsible for processing visual information^24^; and the subcortical network, which plays a role in processing emotions and maintaining consciousness^25^.

Structural networks, on the other hand, have been mostly based on diffusion tensor imaging (DTI) data^26,27^, which allow estimating the physical connections among brain regions, using a technique known as tractography^28,29^. Nevertheless, there have also been a few studies presenting structural brain networks based on cortical thickness^30–35^, volume^36–39^, and morphology^40,41^. The first structural network was discovered by He et al. using cortical thickness data obtained from magnetic resonance images^42^. This network was later shown to coincide with known functional areas of the brain^43^. There is a morphological basis for structural brain networks based on cortical thickness or volume, since the neuronal numbers and layer/tissue architecture can be associated with function^44^.

Brain networks may be studied using a variety of methods, such as seed-based approaches^45^ and independent component analysis (ICA)^46,47^. Nevertheless, graph theory has become the method of choice, since it constitutes a simple but thorough way to represent the brain^48^. Henceforth, whenever we mention "networks", we mean whole-brain networks as in graph theory. In graph models, nodes are usually taken to be brain regions or sensors (such as electrodes in EEG), and links are obtained from the relationship between these nodes. In the case of functional data, these relationships are derived by applying some similarity measure to the nodes’ time series. For example, for fMRI data, the most used similarity measure is Pearson’s correlation, although other measures such as mutual information have also been explored^49^; for EEG data, measures such as coherence^50,51^, phase lag index^52^, and symbolic usual information^7^ have been used. The similarity index allows building the connectivity matrix, which relates every pair of nodes. This matrix can be binarized by applying a threshold to the similarity measure, yielding the adjacency matrix, which states whether two nodes are (1) or not (0) linked. From either the connectivity or the adjacency matrix, measures describing the graph’s topology can be computed. In turn, these measures can be used to characterize the underlying brain (or brains, in the case of group studies)^53^.

Brain networks, both functional and structural, and derived graph measures, have been used to characterize the brain in different situations, such as in normal aging^54,55^, in several types of neurological diseases and conditions (Alzheimer’s^56,57^, epilepsy^58,59^, stroke^60,61^), in the signal generation for brain-computer interfaces^62,63^, among others. Graph measures provide a way to reduce the large amount of information present in brain data to a few useful parameters. However, as mentioned, there have been few studies that have attempted to build networks from structural properties.

Therefore, the main objective of the present work was to investigate the feasibility of obtaining useful information from brain networks derived from structural magnetic resonance (MR) images of healthy individuals, using a different structural property of the regions, namely, texture features based on the gray level co-occurrence matrix (GLCM) method. Texture features have been shown in several studies to be useful for characterizing and differentiating healthy subjects and neurological patients^64,65^. The GLCM, one of the first texture analysis techniques, is a well-established method with several applications, having been successfully used in medical images^66–68^. Therefore, we believe texture-based networks might have the potential to do so and even more. We hypothesized that texture networks for healthy individuals would be able to provide structural relationships between different regions allowing us to characterize this group, particularly regarding age and gender. As a secondary objective, we also aimed to evaluate texture-based structural connections between regions belonging to well-established resting-state functional networks, to see if there were any underlying texture similarities among these areas. The functional networks evaluated were the already mentioned DMN, sensory-motor, attention, visual, and subcortical networks. There are several other resting-state functional brain networks (e.g. limbic, auditory, temporal), some of which may contain some of the regions observed in this article. However, this study was limited to the five networks presented before because they are more well-established and composed of a greater number of regions. Some of the selected networks are even considered core networks according to the proposal of Uddin et al.^69^. Finally, graph metrics associated with texture networks were evaluated for different brain regions, and each region metric was compared to the mean value obtained over all regions, in an attempt to find regions that would stand out in terms of their texture network characteristics. In summary, we wanted to see if texture networks would bring any new, interesting results, in terms of characterization of healthy individuals, because if so, this methodology might also be useful for differentiating neurological patients and aiding with their diagnosis and/or prognosis.

## Subjects, materials, and methods

Structural MR images (T1-weighted) of 760 subjects (mean age 39 ± 14 years; median age 36 ± 14; 300 men) were used in this study. All subjects were over 18 years old, did not have any previous history of neurological illness or brain injury, and signed an informed consent form previous to data acquisition. All experiments were performed in accordance with relevant guidelines and regulations. The study was approved by the Research Ethics Committee of UNICAMP.

Images were acquired in a 3 T MRI scanner (Achieva, Philips, The Netherlands). The pulse sequence was a 3D T1-weighted sequence, with time of repetition TR = 7.1 ms, time to echo TE = 3.2 ms, flip angle of 8°, isotropic voxels of 1.0 × 1.0 × 1.0 mm^3^, and field of view FOV = 240 × 240 mm2.

To explore the structural relationship between different brain regions, the images had to be segmented into regions of interest (ROIs), which was done using the atlas approach. The chosen atlas was the Automated Anatomical Labelling (AAL)^70^, composed of 116 anatomical regions, and included in the PickAtlas software (http://fmri.wfubmc.edu/software/PickAtlas). This atlas corresponds to the most cited paper in the neuroimaging field^71^, and therefore, it was deemed as a good first choice for this exploratory study. Future studies may explore this method with other more modern atlases (e.g. Desikan^72^, Destrieux^73^, and Glaser^74^).

The AAL is in the standard MNI space^75^, therefore, subjects’ images were first converted to this space using the SPM12 software (https://www.fil.ion.ucl.ac.uk/spm/software/spm12/). To do this, the anterior commissure was set to be the origin of the reference system of the images, and these were realigned according to the MNI space orientation. Next, the images were segmented into gray matter, white matter, and cerebrospinal fluid; normalized to the standard MNI space, and registered to the AAL atlas. The brain and intracranial volumes were calculated during this step using the UF2C software^76^. Some of these pre-processing steps involved interpolation methods, which can lead to changes in the image’s texture. When possible, precautions were taken to minimize this effect, such as employing a 4th-degree B-spline, a smoother interpolation method. Finally, the AAL regions were used as masks to identify the regions of interest (ROIs) in the patients' images.

Texture parameters from each ROI were then extracted from the images, using the gray level co-occurrence matrix (GLCM) approach^77^, implemented through homemade Matlab routines. The GLCM is a symmetrical square matrix, where each element (*i*, *j*) represents the (normalized) number of pixel pairs in the ROI with gray levels *i* and *j*, separated by a given distance in a given direction. Usually, the GLCM is computed for 2D images, for distance values ranging from 1 to 5 pixels, and directions horizontal, vertical, and diagonals (45° or 135°). For 3D images, texture parameters from the corresponding 2D slices are combined using simple or weighted average^78–80^.

**Figure 1:**
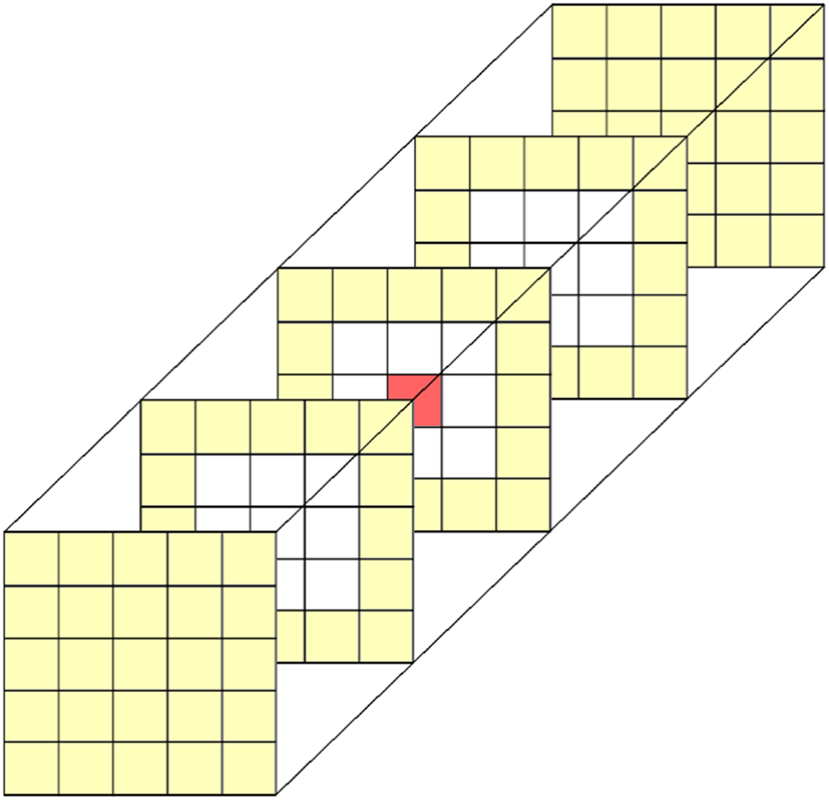
Example of a cubic layer (voxels in yellow) used to compute an isotropic gray level co-occurrence matrix (GLCM) directly from a 3D image.

In this work, an isotropic GLCM (i.e., not considering any particular direction), computed directly from the 3D images, was used. For this, a cubic layer centered in the reference voxel was defined, as outlined in Fig. 1 for a distance of 2 voxels (represented in yellow). In the ROIs border, only voxels contained in the ROI were accounted for the GLCM.

Distance values ranging from 1 to 5 voxels were used so that, for each ROI, five 3D GLCMs were obtained. Initially, GLCMs were calculated for 128 gray levels. This led to very sparse GLCM matrices (~ 90% zero entries for the 1-voxel distance to ~ 87% zero entries for the 5-voxel distance). Therefore, the number of gray levels was lowered to 64, and finally to 32, which decreased sparsity while still keeping some gray-level information (~ 86 to ~ 83% zero entries for the 1 and 5-voxel distance respectively). Although reducing the number of gray levels decreases the sparsity of the GLCM, this also reduces its size, and consequently, the information contained in it. Therefore, it is necessary to balance the trade-off between the information content and the matrix sparsity.

At first, texture parameters from all 116 AAL regions were used to obtain the brain networks. However, the small size of some of these regions led again to an increased number of zeros in their respective GLCMs. Therefore, a selection criterion was applied: regions had to be larger than 900 voxels or, if they were part of a homologous pair, at least one of the regions had to be larger than 1000 voxels. This resulted in a set of 86 AAL regions (these are shown in Table S1 of the Supplementary Material).

**Figure 2:**
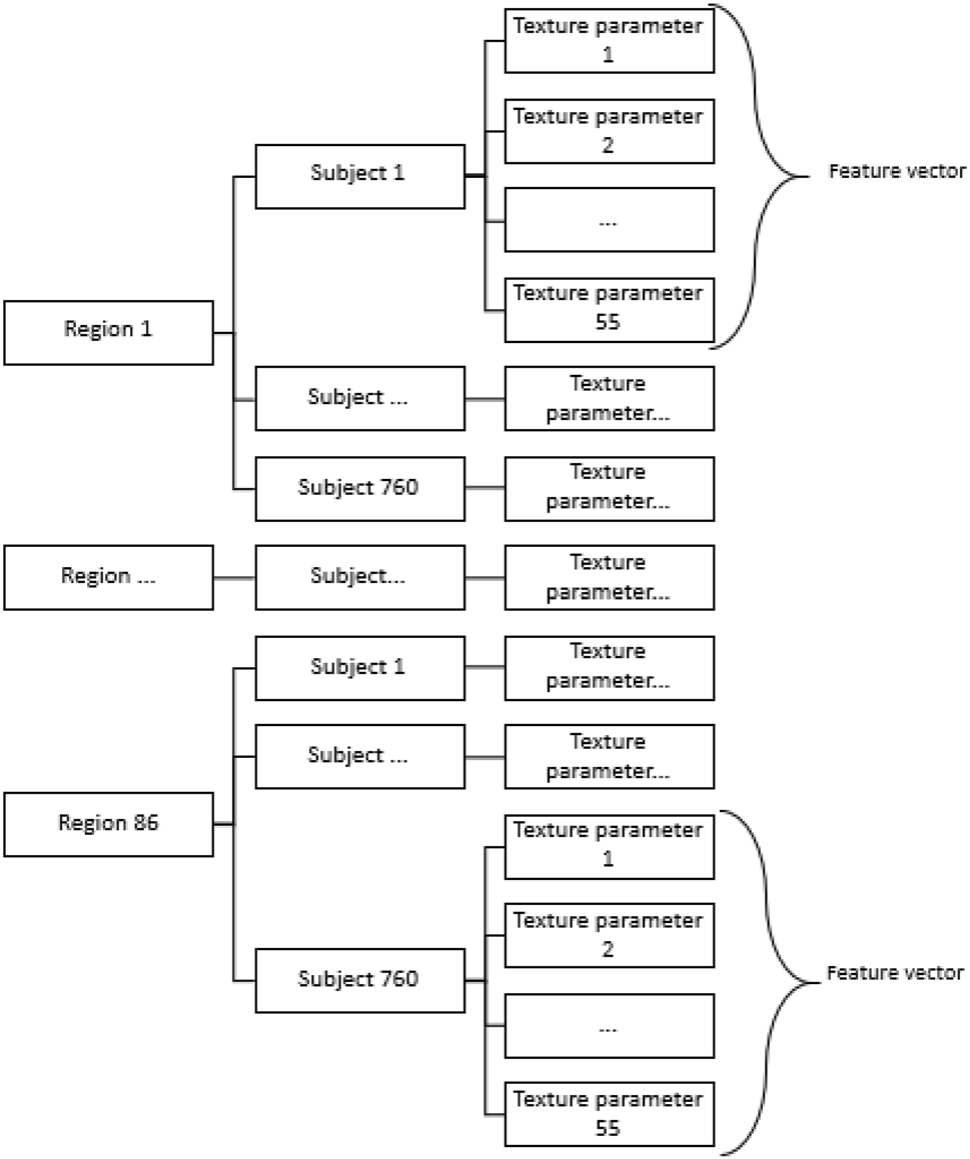
Scheme to obtain the feature vectors.

From each GLCM, 11 texture parameters were computed: uniformity, contrast, correlation, variance, homogeneity, entropy, sum average, sum variance, sum entropy, difference variance, and difference entropy^77^. Therefore, since there were five GLCMs (for distances from 1 to 5 pixels) for each anatomic region, this resulted in a total of 55 parameters per region. Since these parameters have largely different ranges, they were all normalized to be within the [0, 1] range. Finally, a feature vector consisting of these 55 parameters was used to characterize each network node (ROI) of a given individual. Figure 2 shows an explanatory scheme for the generation of the feature vectors.

To compare the feature vectors from every pair of nodes, the inverse of the Euclidean distance was calculated, and those values were normalized to the [0,1] interval. In this way, nodes with more similar texture values (and therefore smaller Euclidian distance between them) would have a stronger link. These values were then employed to generate the brain network links of the weighted graphs of the subjects.

Once the networks were generated, the next step was to compare them. This was done through mathematical measures that describe the topological characteristics of the networks—the network measures^53^. Five network measures were computed for each brain network generated: strength (ST), betweenness centrality (BC), eigenvector centrality (EC), clustering coefficient (CC), and local efficiency (LE). Regarding the measures’ choice, this was driven by two factors: easiness of interpretation and popularity of the measure. The chosen measures have both been used in other works on brain networks using graphs^81–83^ and have a fairly straightforward interpretation.

Four studies were conducted to explore the potential of texture-based networks to characterize a given population. These were:

1. Analysis of structural texture-based networks and comparison with well-established functional networks—For the five functional networks evaluated (DMN, sensory-motor, attention, visual, and subcortical), the mean value of each connection across all individuals and the mean value over all connections and individuals were computed. Then, for each network, the mean connection values (over individuals) were compared with the mean value of all connections. Table S2 in the Supplementary Material shows all the regions belonging to each functional network selected.
2. Comparison between texture-based networks obtained from male and female populations—For each individual brain network, five network measures were extracted—ST, BC, EC, CC, and LE. A statistical test of the ANCOVA type was performed, in which gender was employed as the independent variable and the dependent variables were set as the values of the network measure of each region. Age, brain volume, and intracranial volume were selected as covariates and a Bonferroni correction for multiple comparisons was applied.
3. Analysis of age dependence of texture-based networks—The same five network measures used to evaluate gender differences were extracted from each individual network to investigate age dependence—ST, BC, EC, CC, and LE. A statistical test of the ANCOVA type was performed, in which age was selected as the independent variable and the dependent variable was the network measure of each region. Sex, brain volume, and intracranial volume were selected as covariates, and correction for multiple comparisons was performed using Bonferroni.
4. Analysis of network measure variation for different brain regions—The same five network measures were extracted from each individual network—ST, BC, EC, CC, and LE. For each network measure, the mean of each region’s network measure over the entire population was calculated, as well as the global mean (over all regions and individuals). Then, the difference between each region’s mean and the global mean was computed.

## Results and discussion

### Analysis of structural texture-based networks and comparison with well-established functional networks

The similarity measure was calculated between all regions in the network and the mean of these measures was computed. We evaluated the number of edges between the N nodes of each functional network as N*(N-1)/2. We then looked at the value of the similarity measure corresponding to each edge and compared it to the previous mean. Table 1 shows the number of edges that are greater than the mean, as well as the number of edges that are at least one standard deviation greater than the mean.

**Table 1:** Number of possible connections between the regions comprising each evaluated brain functional network; and number and percentage of connections with a mean value of similarity measure between regions greater than the mean value of all connections.

Network	Possible connections	Greater than mean		1 standard deviation greater than mean	
		Connections	%	Connections	%
Default mode	66	45	68.2	5	7.6
Sensory-motor	153	68	44.4	16	10.5
Attention	153	110	71.9	23	15.0
Visual	91	63	69.2	12	13.2
Subcortical	91	19	20.9	3	3.3

Although three out of the five evaluated functional networks presented around 70% of their texture-based connections stronger than the mean connection value, when we look at how strong these values were, we see that only between 7 and 15% of the values were actually larger than one standard deviation. Therefore, this seems to indicate that there isn’t a morphological texture-based subsidy for these functional networks. This result differs from other results in the literature, such as the study by Park and colleagues, who found a high agreement between brain parcellations based on fMRI networks and cortical thickness networks for the medial frontal cortex^84^, or the study by Chen and coworkers, who found that functional domains such as auditory/language, strategic/executive, sensorimotor, visual, and mnemonic processing had a close overlap with cortical thickness network modules^43^. However, these differences with our results can be explained because although texture measures are expected to reflect the underlying tissue structure, these are usually evaluated from volumetric ROIs that encompass sulci and gyri, and therefore manifest different morphological properties than those disclosed by cortical thickness.

### Comparison between texture-based networks obtained from male and female populations

Among the 86 regions selected, two regions showed significant differences (ANCOVA, *p* < 0.05) among male and female populations for all five network measures, and 26 regions showed significant differences for four network measures. The remaining regions presented significant differences as follows: 9 regions for three network measures, 10 regions for two network measures, 28 regions for a single network measure, and 11 regions presented no significant differences for any network measure. Table 2 shows the regions for which at least four network measures were significantly different (ANCOVA, *p* < 0.05) among male and female populations. Table S3 in the Supplementary Material shows all the regions with at least one significantly different network measure.

**Table 2:** Regions that had at least four significant (ANCOVA, *p* < 0.05) network measures regarding differences between male and female populations.

Region	ST	BC	EC	CC	LE
Angular_R	**0.000**	–	**0.000**	**0.000**	**0.000**
Cerebellum_4_5_L	**0.000**	–	**0.000**	**0.007**	**0.009**
Cerebellum_4_5_R	**0.003**	–	**0.000**	**0.016**	**0.019**
Cerebellum_Crus2_L	**0.000**	–	**0.002**	**0.000**	**0.000**
Cerebellum_Crus2_R	**0.000**	–	**0.000**	**0.000**	**0.000**
Frontal_Inf_Oper_L	**0.001**	0.506	**0.005**	**0.001**	**0.001**
Frontal_Inf_Oper_R	**0.000**	0.945	**0.000**	**0.000**	**0.000**
Frontal_Inf_Tri_R	**0.000**	**0.017**	**0.000**	**0.000**	**0.000**
Frontal_Mid_L	**0.000**	–	**0.000**	**0.000**	**0.000**
Frontal_Mid_R	**0.000**	–	**0.000**	**0.000**	**0.000**
Frontal_Sup_L	**0.003**	0.102	**0.004**	**0.001**	**0.001**
Frontal_Sup_Medial_L	**0.000**	0.151	**0.000**	**0.000**	**0.000**
Frontal_Sup_Orb_L	**0.001**	0.936	**0.003**	**0.001**	**0.001**
Frontal_Sup_R	**0.001**	0.709	**0.003**	**0.000**	**0.001**
Paracentral_Lobule_R	**0.001**	0.877	**0.001**	**0.002**	**0.002**
Parietal_Inf_L	**0.000**	0.374	**0.000**	**0.002**	**0.002**
Parietal_Inf_R	**0.001**	0.537	**0.002**	**0.001**	**0.001**
Parietal_Sup_L	**0.006**	0.985	**0.008**	**0.003**	**0.003**
Parietal_Sup_R	**0.008**	0.154	**0.008**	**0.004**	**0.005**
Postcentral_L	**0.003**	0.808	**0.001**	**0.002**	**0.003**
Postcentral_R	**0.000**	0.081	**0.000**	**0.000**	**0.000**
Precentral_L	**0.000**	0.752	**0.000**	**0.000**	**0.000**
Precentral_R	**0.000**	0.877	**0.000**	**0.000**	**0.000**
Precuneus_L	**0.006**	–	**0.004**	**0.004**	**0.004**
Supp_Motor_Area_L	**0.008**	**0.047**	**0.016**	**0.006**	**0.006**
Supra_Marginal_R	**0.000**	0.714	**0.000**	**0.000**	**0.000**
Temporal_Pole_Mid_R	**0.000**	0.826	**0.050**	**0.001**	**0.001**
Temporal_Pole_Sup_R	**0.000**	–	**0.000**	**0.000**	**0.000**

*P*-values smaller than 0.05 are marked in bold font.

In summary, we obtained network measures that were significantly different among men and women for most regions (75 out of 86). This indicates a strong relationship between sex and texture connections. In decreasing order, 62 regions showed significant differences for the EC, 43 for ST, 41 for both CC and LE, and 2 for BC.

The regions for which all network measures were significant were the right triangular part of the inferior frontal gyrus (Frontal Inf Tri) and the left supplementary motor area (Supp Motor Area). Along with the opercular part, the right Frontal Inf Tri is associated with Broca's area, which is involved in speech production. Specifically, Frontal Inf Tri is involved with the semantic processing of language and non-verbal communication such as gesticulation and facial expression^85^. Supp Motor Area has a role in the preparation of voluntary movements and the temporal organization of sequential movements^86^. For these regions, ST, EC, CC, and LE had larger values for men than for women. For BC, Supp Motor Area had larger values for men than for women, while the opposite happened for Frontal Inf Tri.

Since ST is the sum of connections of a node, this measures how strongly connected this node is. EC measures a node’s influence in the network, while CC points to a node’s tendency to form clusters. Both LE and CC analyze the shortest paths that connect nodes. LE measures the minimum number of nodes necessary for connecting a pair of nodes, while CC measures the number of shortest paths a node is part of. In the context of texture, this implies that both the right Frontal Inf Tri and left Supp Motor Area have a texture that is highly similar to the surrounding regions, forming a texture cluster with them. These regions, however, are not similar in texture among themselves. This happened more for men than for women. Conversely, BC measures how much a node behaves as a hub, i.e., how much it intermediates relationships among different parts of the network. Thus, considering texture properties, the right Frontal Inf Tri behaves more as hubs for women than for men, while the left Supp Motor Area behaves more as hubs for men than for women.

In addition, nine pairs of homologous regions (lobules IV and V of the cerebellar hemisphere (Cerebellum 4 5), crus II of the cerebellar hemisphere (Cerebellum Crus2), opercular part of the inferior frontal gyrus (Frontal Inf Oper), middle frontal gyrus (Frontal Mid), dorsolateral superior frontal gyrus (Frontal Sup), inferior parietal, but supramarginal and angular gyri (Parietal Inf ), superior parietal gyrus (Parietal Sup), postcentral gyrus (Postcentral), and precentral gyrus (Precentral)), five regions in the right hemisphere (angular gyrus (Angular), paracentral lobule (Paracentral Lobule), supramarginal gyrus (Supra Marginal), middle temporal gyrus of the temporal pole (Temporal Pole Mid), and superior temporal gyrus of the temporal pole (Temporal Pole Sup)) and three regions in the left hemisphere (medial superior frontal gyrus (Frontal Sup Medial), orbital part of the superior frontal gyrus (Frontal Sup Orb), and Precuneus (Precuneus)) were significant for four network measures. As previously, for these regions (for the left and right Cerebellum 4 5), ST, EC, CC, and LE had larger values for men (women) than for women (men). This indicates these regions share texture similarities with other regions, with which they tend to cluster.

Regarding gender, this suggests the texture of these regions (of the Cerebellum 4 5) is more similar to their neighboring regions for male (female) individuals rather than females (males). These regions (the Cerebellum 4 5) also have a higher tendency to form texture clusters for males (females) than for females (males).

### Analysis of age dependence of texture-based networks

Among the 86 regions selected, one region showed significant differences (ANCOVA, *p* < 0.05) for the independent variable age (see Table 3) for all five network measures, and 11 regions showed significant differences for four network measures. There were significant differences for three network measures in two regions, for two network measures in five regions, for a single network measure in 25 regions, and no significant difference for any network measure in 42 regions. Table 4 shows the AAL regions with at least four significant R^2^ values, obtained for the linear regression of the network measures considering the independent variable age. Table S4 (in the Supplementary Material) shows the AAL regions with at least one significant R^2^ value.

**Table 3:** Participants’ age distribution.

Age interval	Number of individuals
18–30	281
31–45	209
46–60	194
61–75	63
76–90	13

**Table 4:** R squared and p-values obtained from ANCOVA for the independent variable age for the regions that had at least four significant (*p* < 0.05) network measures.

	ST		BC		EC		CC		LE	
	R squared	Sig.	R squared	Sig.	R squared	Sig.	R squared	Sig.	R squared	Sig.
Caudate_L	0.181	**0.038**	0.082	0.574	0.220	**0.000**	0.187	**0.042**	0.182	**0.049**
Caudate_R	*0.291*	**0.000**	*–*	–	*0.320*	**0.000**	*0.294*	**0.000**	*0.289*	**0.001**
Cerebellum_6_L	0.131	**0.019**	*–*	–	0.160	**0.013**	0.128	**0.034**	0.126	**0.039**
Cingulum_Ant_L	0.226	**0.001**	*–*	–	*0.358*	**0.000**	0.196	**0.011**	0.189	**0.015**
Frontal_Inf_Tri_R	*0.263*	**0.003**	0.110	0.952	*0.312*	**0.000**	0.241	**0.031**	0.236	**0.037**
Frontal_Sup_L	0.129	**0.017**	0.091	0.580	0.135	**0.005**	0.131	**0.043**	0.130	**0.045**
Insula_L	0.250	**0.000**	*–*	–	*0.383*	**0.000**	0.236	**0.000**	0.228	**0.000**
Insula_R	*0.266*	**0.000**	*–*	–	*0.442*	**0.000**	0.245	**0.000**	0.237	**0.000**
Occipital_Sup_L	0.149	**0.001**	*0.299*	**0.000**	0.167	**0.000**	0.141	**0.010**	0.138	**0.012**
Occipital_Sup_R	0.151	**0.005**	0.046	1.000	0.198	**0.000**	0.143	**0.030**	0.140	**0.035**
Thalamus_L	*0.289*	**0.003**	0.117	0.688	*0.359*	**0.003**	*0.320*	**0.005**	*0.313*	**0.007**
Thalamus_R	*0.284*	**0.006**	0.137	0.833	*0.364*	**0.000**	*0.326*	**0.015**	*0.319*	**0.019**

*p-*values smaller than 0.05 are marked in bold font, R^2^ values greater than 0.250 are marked in italic font.

Forty-four (out of 86) regions had network measures that presented a significant dependence on age. In decreasing order, there were significant differences for EC in 40 regions, ST in 17, CC and LE in 12, and BC in 9. From these, 12 regions had at least four significant network measures (out of five tested measures). There were four pairs of homologous regions (caudate nuclei (Caudate), insula (Insula), superior occipital gyri (Occipital Sup), and thalami (Thalamus)), one region in the right hemisphere (triangular part of the inferior frontal gyrus (Frontal Inf Tri)), and three regions in the left hemisphere (lobule VI of cerebellar hemisphere (Cerebellum 6), anterior cingulate & paracingulate gyrus (Cingulum Ant), and dorsolateral superior frontal gyrus).

**Figure 3:**
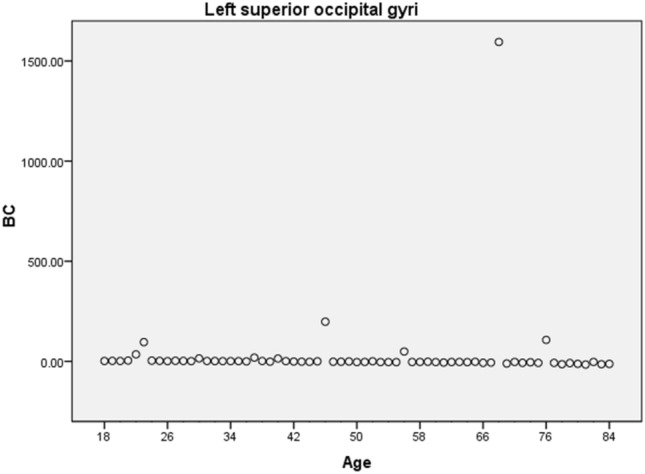
Age versus BC for the left Occipital Sup. The plot shows that even though BC presented values of R^2^ greater than 0.250 and *p*-values lower than 0.05, those are not meaningful since only a few of the data points are non-null.

The two largest values of R^2^ obtained were 0.503 for the right hippocampus and 0.502 for the right supramarginal gyrus (see Table S4 in the Supplementary Material), all referring to the BC network measure. However, despite the corresponding p-values being significant (*p* < 0.05), the plot of age versus BC (Fig. 3) shows this result is not meaningful, since the majority of data points are zero.

**Figure 4:**
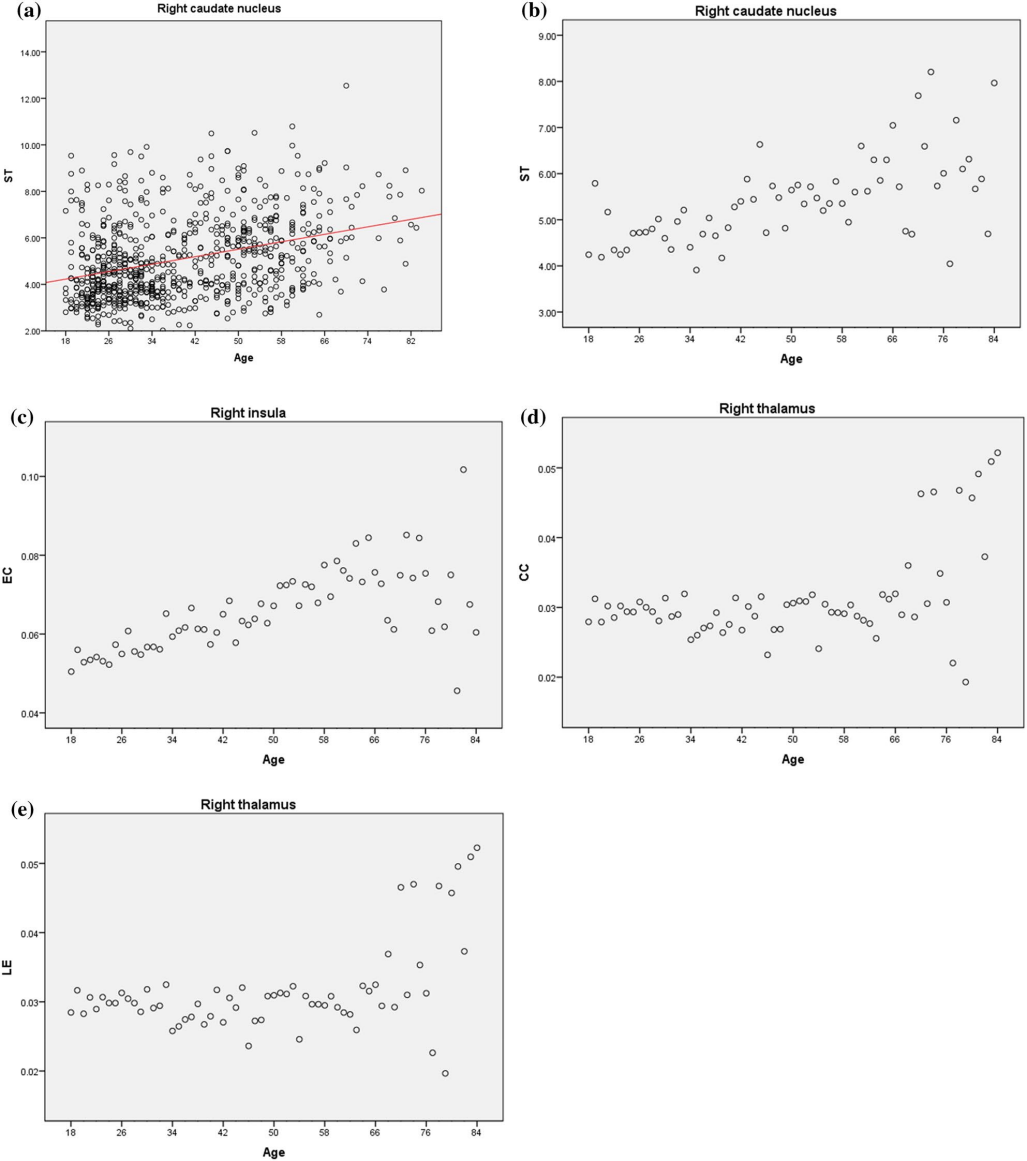
Age versus network measure plots. ST dispersion plot (**a**) for the right Caudate with linear adjustment (red line). Estimated marginal means plots of ST (**b**) for the right Caudate, EC (**c**) for the right Insula, and CC (**d**) and LE (**e**) for the right Thalamus.

On the other hand, the other network measures did show meaningful results (Fig. 4), but the R^2^ values obtained in these cases were smaller than 0.5, meaning that the relationship between texture and age is not linear.

For the ST, CC, and LE, all regions showed an increase with age. For the EC, only the left Frontal Sup showed a decrease with age, while all the other regions showed an increase.

An increase with age of the ST, CC, EC, and LE suggests the texture of these regions becomes more similar to their neighboring regions, while at the same time, developing a progressively higher tendency to form texture clusters. This increase in their similarity can be a result of brain degeneration, which agrees with the reported age-related reduction in brain volume^87^.

Thus, it was not possible to establish a linear relationship between texture and age. Yet, this does not mean that there is no relation between these factors because several significant values were obtained.

### Analysis of network measure variation for different brain regions

The same brain network measures used in the previous studies were computed from the networks: ST, BC, EC, CC, and LE. For each region (node), the network measures were averaged over the entire population. Also, a global mean value (over all regions) was calculated for each graph measure. Finally, the difference between each region’s graph measure and the global mean was calculated, in units of standard deviation, and a threshold of 1.5 was then applied. Table 5 shows the results for which this difference is greater than this threshold.

**Table 5:** Difference between each region’s network measure and the global mean of the measure (in standard deviation units) for the individual networks.

Region	Network measure				
	ST	BC	EC	CC	LE
L putamen	− 2.102	4.103	− 2.285	− 2.357	− 2.349
R putamen	− 2.099	3.103	− 2.278	− 2.345	− 2.336
L thalamus	− 1.978	–	− 2.170	− 2.138	− 2.128
R thalamus	− 1.989	1.713	− 2.182	− 2.166	− 2.156
R caudate	–	–	− 1.562	–	–

The putamen and the thalamus presented decreases in ST, EC, CC, and LE, compared to the global mean for these measures. Since these measures are related to: similarity of the region with directly connected regions (ST); similarity among neighbor regions excluding the region itself (CC); similarity of the region with indirectly connected regions (LE); and similarity among the measures of the region with those of directly connected regions (EC)—this result implies that structurally (texture-wise), these regions are very unique compared to other analyzed regions. I.e., these regions' textures are quite different from the regions connected, directly or indirectly, to them. On the other hand, these same regions presented an increase in BC, meaning that, structurally (texture-wise), they play an important role as hubs.

The putamen regulates movements at various stages (e.g. preparation and execution) and influences several types of learning^88^. The thalamus’ main function is relaying sensory signals, including motor signals, to the cerebral cortex^89^. It is also responsible for the regulation of consciousness, sleep, and alertness^90^. Given their very specialized functions, it seems appropriate that they present these very differentiated measures in the texture networks.

Lastly, the caudate also presented a decrease in eigenvector centrality. The caudate nucleus is associated with motor processes, but with important roles in procedural learning, associative learning, and inhibitory control of actions^91,92^. A decrease in this network measure compared to the regions’ average means that, texture-wise, this region has a lesser influence on the network than other regions.

### Work limitations

The approach employed in this work presented interpretation difficulties since some network measures are hard to decipher based on texture (e.g., efficiency, eigenvector centrality).

Other limitations can be related to the image’s preprocessing performed, which relies on interpolation methods, which could lead to alterations in the image’s texture. This could be prevented by analyzing those images in the native space. Another issue faced is related to the number of gray levels. A higher number could lead to more information, but on the other hand, it means a greater number of zeros in the GLCM, implying less significant data. This was solved by reducing the number of gray levels for the GLCM.

## Conclusion

In this work, we attempted to use texture analysis to generate brain networks based on structural properties of magnetic resonance images. To the best of our knowledge, this is the first work to use image texture properties to build brain networks.

We sought to evaluate the structural connections between regions of well-established functional networks, changes in the brain networks due to gender, and the dependence of the networks with age. We also employed graph theory to attempt to characterize healthy individuals. We were able to extract meaningful information from the texture-based networks, thus proving its usefulness.

The comparison study between the structural texture-based networks and some well-established functional networks did not allow to establish a morphological relation between the texture-based networks and the functional networks.

The gender comparison study showed significant differences in the network measures for most (75/86) regions investigated. In fact, network measures obtained suggested higher similarity among neighboring regions and a higher tendency to form texture clusters for male than for female individuals, except for the Cerebellum 4 5, which presents higher similarity among neighboring regions and a higher tendency to form texture clusters for female than for male individuals. Also, this analysis found that the left Supp Motor Area (right Frontal Inf Tri) was more likely to behave as a texture hub for male (female) individuals.

Regarding the age study, around half (44/86) of the selected regions yielded significant nonlinear relations with age. Network measure changes with age for the majority of these regions showed an increase in texture similarity among them, possibly related to degeneration of the underlying tissues.

The graph metrics’ study showed that the thalamus and the putamen display weaker connections to other regions, but they function as hubs. Therefore, they have, texture-wise, quite a unique structure, which seems appropriate, due to their respective roles.

All analyses performed in this work were based on T1-weighted structural magnetic images. For future studies, T2-weighted resonance images or even multimodal images could be analyzed. This work used the co-occurrence matrix method for texture analysis, but future works could employ other techniques (e.g., wavelets^93^ or local binary patterns^94,95^). The atlas chosen for the parcellation contained many small regions—due to the great number of subdivisions—and could be replaced by an atlas with fewer (and therefore bigger) regions. On the other hand, the atlas-based parcellation could also be replaced with a learning-based parcellation. Furthermore, machine learning algorithms could be applied to the data obtained to extract meaningful information.

This work’s methodology was applied to healthy individuals. Further investigation is required to determine if it is possible to employ these methods for patients with anatomical alterations. Indeed, the next step for this work is to apply these techniques to patients with different brain diseases/conditions (e.g. Alzheimer’s disease), to investigate the ability of the proposed method to produce biomarkers for these pathologies.

### Supplementary Information

Supplementary Information.

## Data Availability

The datasets generated during and/or analyzed during the current study are available from the corresponding author on reasonable request.
