# Supplementary material for: Texture-based brain networks for characterization of healthy subjects from MRI
Source: Sci Rep. 2023 Sep 29;13:16421. doi: 10.1038/s41598-023-43544-6 (PMC10541866; doi:10.1038/s41598-023-43544-6)
Supplement: Supplementary file 1 — Supplementary Information. [file 41598_2023_43544_MOESM1_ESM.docx]

**Texture-based brain networks for characterization of healthy subjects from MRI**

**Supplementary Material**

**Table S1.** AAL anatomical regions that follow the size criteria and their respective size, in voxels

| AAL region | | Anatomical region | Size (voxels) | |
| --- | --- | --- | --- | --- |
| Angular L | | Angular gyrus | 1173 | |
| Angular R | |  | 1752 | |
| Calcarine L | | Calcarine fissure and surrounding cortex | 2258 | |
| Calcarine R | |  | 1861 | |
| Caudate L | | Caudate nucleus | 962 | |
| Caudate R | |  | 994 | |
| Cerebellum 4 5 L | | Lobule IV, V of the cerebellar hemisphere | 1125 | |
| Cerebellum 4 5 R | |  | 861 | |
| Cerebellum 6 L | | Lobule VI of the cerebellar hemisphere | 1694 | |
| Cerebellum 6 R | |  | 1795 | |
| Cerebellum 8 L | | Lobule VIII of the cerebellar hemisphere | 1887 | |
| Cerebellum 8 R | |  | 2308 | |
| Cerebellum Crus1 L | | Crus I of the cerebellar hemisphere | 2603 | |
| Cerebellum Crus1 R | |  | 2648 | |
| Cerebellum Crus2 L | | Crus II of the cerebellar hemisphere | 1894 | |
| Cerebellum Crus2 R | |  | 2117 | |
| Cingulum Ant L | | Anterior cingulate and paracingulate gyri | 1400 | |
| Cingulum Ant R | |  | 1313 | |
| Cingulum Mid L | | Median cingulate and paracingulate gyri | 1941 | |
| Cingulum Mid R | |  | 2203 | |
| Cuneus L | | Cuneus | 1526 | |
| Cuneus R | |  | 1424 | |
| Frontal Inf Oper L | | Inferior frontal gyrus, opercular part | 1038 | |
| Frontal Inf Oper R | |  | 1399 | |
| Frontal Inf Orb L | | Inferior frontal gyrus, orbital part | 1690 | |
| Frontal Inf Orb R | |  | 1707 | |
| Frontal Inf Tri L | | Inferior frontal gyrus, triangular part | 2529 | |
| Frontal Inf Tri R | |  | 2151 | |
| Frontal Mid L | | Middle frontal gyrus | 4863 | |
| Frontal Mid R | |  | 5104 | |
| Frontal Mid Orb L | | Middle frontal gyrus, orbital part | 888 | |
| Frontal Mid Orb R | |  | 1015 | |
| Frontal Sup L | | Superior frontal gyrus, dorsolateral | 3599 | |
| Frontal Sup R | |  | 4056 | |
| Frontal Sup Medial L | Superior frontal gyrus, medial | | | 2992 |
| Frontal Sup Medial R |  |  |  | 2134 |
| Frontal Sup Orb L | Superior frontal gyrus, orbital part | | | 963 |
| Frontal Sup Orb R |  |  |  | 997 |
| Fusiform L | Fusiform gyrus | | | 2310 |
| Fusiform R |  |  |  | 2518 |
| Hippocampus L | Hippocampus | | | 932 |
| Hippocampus R |  |  |  | 946 |
| Insula L | Insula | | | 1858 |
| Insula R |  |  |  | 1770 |
| Lingual L | Lingual gyrus | | | 2095 |
| Lingual R |  |  |  | 2300 |
| Occipital Inf L | Inferior occipital gyrus | | | 941 |
| Occipital Inf R |  |  |  | 989 |
| Occipital Mid L | Middle occipital gyrus | | | 3270 |
| Occipital Mid R |  |  |  | 2098 |
| Occipital Sup L | Superior occipital gyrus | | | 1366 |
| Occipital Sup R |  |  |  | 1413 |
| Paracentral Lobule L | Paracentral lobule | | | 1349 |
| Paracentral Lobule R |  |  |  | 836 |
| Para Hippocampal L | Parahippocampal gyrus | | | 978 |
| Para Hippocampal R |  |  |  | 1132 |
| Parietal Inf L | Inferior parietal, but supramarginal and angular gyri | | | 2447 |
| Parietal Inf R |  |  |  | 1345 |
| Parietal Sup L | Superior parietal gyrus | | | 2065 |
| Parietal Sup R |  |  |  | 2222 |
| Postcentral L | Postcentral gyrus | | | 3892 |
| Postcentral R |  |  |  | 3823 |
| Precentral L | Precental gyrus | | | 3526 |
| Precentral R |  |  |  | 3381 |
| Precuneus L | Precuneus | | | 3528 |
| Precuneus R |  |  |  | 3265 |
| Putamen L | Lenticular nucleus, putamen | | | 1009 |
| Putamen R |  |  |  | 1064 |
| Rolandic Oper L | Rolandic operculum | | | 990 |
| Rolandic Oper R |  |  |  | 1331 |
| Supp Motor Area L | Supplementary motor area | | | 2147 |
| Supp Motor Area R |  |  |  | 2371 |
| Supra Marginal L | Supramarginal gyrus | | | 1256 |
| Supra Marginal R |  |  |  | 1974 |
| Temporal Inf L | Inferior temporal gyrus | | | 3200 |
| Temporal Inf R |  |  |  | 3557 |
| Temporal Mid L | Middle temporal gyrus | | | 4942 |
| Temporal Mid R |  |  |  | 4409 |
| Temporal Pole Mid L | Temporal pole: middle temporal gyrus | | | 755 |
| Temporal Pole Mid R |  |  |  | 1187 |
| Temporal Pole Sup L | Temporal pole: superior temporal gyrus | | | 1285 |
| Temporal Pole Sup R |  |  |  | 1338 |
| Temporal Sup L | Superior temporal gyrus | | | 2296 |
| Temporal Sup R |  |  |  | 3141 |
| Thalamus L | Thalamus | | | 1100 |
| Thalamus R |  |  |  | 1057 |

**Table S2.** Anatomical regions that belong to one of the five selected functional networks – default mode, sensory-motor, attention, visual, and subcortical.

| Brain region | Brain network |
| --- | --- |
| Angular L | Attention |
| Angular R | Attention |
| Calcarine L | Visual |
| Calcarine R | Visual |
| Caudate L | Subcortical |
| Caudate R | Subcortical |
| Cingulum Ant L | Default Mode |
| Cingulum Ant R | Default Mode |
| Cingulum Mid L | Subcortical |
| Cingulum Mid R | Subcortical |
| Cuneus L | Visual |
| Cuneus R | Visual |
| Frontal Inf Oper L | Attention |
| Frontal Inf Oper R | Attention |
| Frontal Inf Orb L | Attention |
| Frontal Inf Orb R | Attention |
| Frontal Inf Tri L | Attention |
| Frontal Inf Tri R | Attention |
| Frontal Mid L | Attention |
| Frontal Mid Orb L | Attention |
| Frontal Mid Orb R | Attention |
| Frontal Mid R | Attention |
| Frontal Sup L | Default Mode |
| Frontal Sup Medial L | Default Mode |
| Frontal Sup Medial R | Default Mode |
| Frontal Sup Orb L | Attention |
| Frontal Sup Orb R | Default Mode |
| Frontal Sup R | Default Mode |
| Fusiform L | Visual |
| Fusiform R | Visual |
| Hippocampus L | Subcortical |
| Hippocampus R | Subcortical |
| Insula L | Sensory-motor |
| Insula R | Sensory-motor |
| Lingual L | Visual |
| Lingual R | Visual |
| Occipital Inf L | Visual |
| Occipital Inf R | Visual |
| Occipital Mid L | Visual |
| Occipital Mid R | Visual |
| Occipital Sup L | Visual |
| Occipital Sup R | Visual |
| Paracentral Lobule L | Sensory-motor |
| Paracentral Lobule R | Sensory-motor |
| Para Hippocampal L | Subcortical |
| Para Hippocampal R | Subcortical |
| Parietal Inf L | Attention |
| Parietal Inf R | Attention |
| Parietal Sup L | Sensory-motor |
| Parietal Sup R | Sensory-motor |
| Postcentral L | Sensory-motor |
| Postcentral R | Sensory-motor |
| Precentral L | Sensory-motor |
| Precentral R | Sensory-motor |
| Precuneus L | Default Mode |
| Precuneus R | Default Mode |
| Putamen L | Subcortical |
| Putamen R | Subcortical |
| Rolandic Oper L | Sensory-motor |
| Rolandic Oper R | Sensory-motor |
| Supp Motor Area L | Attention |
| Supp Motor Area R | Sensory-motor |
| Supra Marginal L | Sensory-motor |
| Supra Marginal R | Sensory-motor |
| Temporal Inf L | Attention |
| Temporal Inf R | Default Mode |
| Temporal Mid L | Default Mode |
| Temporal Mid R | Default Mode |
| Temporal Pole Mid L | Subcortical |
| Temporal Pole Mid R | Subcortical |
| Temporal Pole Sup L | Attention |
| Temporal Pole Sup R | Sensory-motor |
| Temporal Sup L | Sensory-motor |
| Temporal Sup R | Sensory-motor |
| Thalamus L | Subcortical |
| Thalamus R | Subcortical |

**Table S3.** Regions that had at least one significant (ANCOVA, p<0.05) network measure regarding differences between male and female populations. P-values smaller than 0.05 are marked in bold font.

| AAL Region | ST | BC | EC | CC | LE |
| --- | --- | --- | --- | --- | --- |
| Angular_L | 0,638 | - | **0,000** | 0,238 | 0,240 |
| Angular_R | **0,000** | - | **0,000** | **0,000** | **0,000** |
| Calcarine_L | 0,583 | - | **0,000** | 0,178 | 0,176 |
| Calcarine_R | 0,367 | 0,916 | **0,000** | 0,720 | 0,705 |
| Caudate_L | **0,036** | 0,663 | 0,897 | **0,047** | **0,045** |
| Caudate_R | **0,003** | - | 0,199 | **0,008** | **0,007** |
| Cerebellum_4_5_L | **0,000** | - | **0,000** | **0,007** | **0,009** |
| Cerebellum_4_5_R | **0,003** | - | **0,000** | **0,016** | **0,019** |
| Cerebellum_6_L | **0,038** | - | **0,000** | 0,086 | 0,100 |
| Cerebellum_6_R | **0,019** | - | **0,000** | 0,117 | 0,132 |
| Cerebellum_8_L | 0,288 | - | **0,000** | 0,605 | 0,641 |
| Cerebellum_8_R | 0,520 | - | **0,006** | 0,415 | 0,400 |
| Cerebellum_Crus1_L | 0,784 | 0,584 | **0,018** | 0,256 | 0,253 |
| Cerebellum_Crus2_L | **0,000** | - | **0,002** | **0,000** | **0,000** |
| Cerebellum_Crus2_R | **0,000** | - | **0,000** | **0,000** | **0,000** |
| Cingulum_Ant_L | 0,500 | - | **0,011** | 0,277 | 0,274 |
| Cingulum_Ant_R | 0,287 | - | **0,047** | 0,085 | 0,085 |
| Cingulum_Mid_L | 0,770 | - | **0,000** | 0,341 | 0,337 |
| Cingulum_Mid_R | 0,189 | - | **0,000** | 0,960 | 0,941 |
| Cuneus_L | **0,008** | - | 0,225 | **0,008** | **0,008** |
| Cuneus_R | 0,722 | 0,430 | **0,009** | 0,412 | 0,417 |
| Frontal_Inf_Oper_L | **0,001** | 0,506 | **0,005** | **0,001** | **0,001** |
| Frontal_Inf_Oper_R | **0,000** | 0,945 | **0,000** | **0,000** | **0,000** |
| Frontal_Inf_Orb_L | 0,648 | - | **0,000** | 0,150 | 0,150 |
| Frontal_Inf_Orb_R | 0,061 | - | 0,989 | **0,026** | **0,028** |
| Frontal_Inf_Tri_R | **0,000** | **0,017** | **0,000** | **0,000** | **0,000** |
| Frontal_Mid_L | **0,000** | - | **0,000** | **0,000** | **0,000** |
| Frontal_Mid_Orb_L | 0,300 | 0,163 | **0,000** | 0,949 | 0,951 |
| Frontal_Mid_Orb_R | **0,027** | 0,152 | **0,000** | 0,335 | 0,341 |
| Frontal_Mid_R | **0,000** | - | **0,000** | **0,000** | **0,000** |
| Frontal_Sup_L | **0,003** | 0,102 | **0,004** | **0,001** | **0,001** |
| Frontal_Sup_Medial_L | **0,000** | 0,151 | **0,000** | **0,000** | **0,000** |
| Frontal_Sup_Medial_R | **0,037** | - | 0,441 | **0,041** | **0,044** |
| Frontal_Sup_Orb_L | **0,001** | 0,936 | **0,003** | **0,001** | **0,001** |
| Frontal_Sup_Orb_R | 0,421 | 0,266 | **0,045** | 0,198 | 0,205 |
| Frontal_Sup_R | **0,001** | 0,709 | **0,003** | **0,000** | **0,001** |
| Fusiform_L | **0,039** | - | **0,000** | 0,324 | 0,351 |
| Fusiform_R | 0,108 | - | **0,000** | 0,584 | 0,614 |
| Hippocampus_L | 0,317 | - | **0,000** | 0,663 | 0,695 |
| Hippocampus_R | **0,049** | 0,631 | **0,000** | 0,377 | 0,401 |
| Insula_L | 0,934 | - | **0,000** | 0,604 | 0,586 |
| Insula_R | **0,026** | - | **0,000** | 0,206 | 0,226 |
| Lingual_L | 0,728 | - | **0,000** | 0,246 | 0,240 |
| Lingual_R | 0,932 | - | **0,000** | 0,471 | 0,462 |
| Occipital_Inf_L | **0,004** | - | 0,129 | **0,002** | **0,002** |
| Occipital_Inf_R | 0,606 | 0,135 | **0,034** | 0,332 | 0,341 |
| Occipital_Mid_L | 0,100 | - | 0,272 | **0,046** | **0,049** |
| Occipital_Mid_R | **0,017** | - | 0,253 | **0,020** | **0,022** |
| Occipital_Sup_R | **0,009** | 0,270 | 0,060 | **0,008** | **0,009** |
| Paracentral_Lobule_R | **0,001** | 0,877 | **0,001** | **0,002** | **0,002** |
| ParaHippocampal_R | 0,550 | - | **0,009** | 0,264 | 0,256 |
| Parietal_Inf_L | **0,000** | 0,374 | **0,000** | **0,002** | **0,002** |
| Parietal_Inf_R | **0,001** | 0,537 | **0,002** | **0,001** | **0,001** |
| Parietal_Sup_L | **0,006** | 0,985 | **0,008** | **0,003** | **0,003** |
| Parietal_Sup_R | **0,008** | 0,154 | **0,008** | **0,004** | **0,005** |
| Postcentral_L | **0,003** | 0,808 | **0,001** | **0,002** | **0,003** |
| Postcentral_R | **0,000** | 0,081 | **0,000** | **0,000** | **0,000** |
| Precentral_L | **0,000** | 0,752 | **0,000** | **0,000** | **0,000** |
| Precentral_R | **0,000** | 0,877 | **0,000** | **0,000** | **0,000** |
| Precuneus_L | **0,006** | - | **0,004** | **0,004** | **0,004** |
| Precuneus_R | 0,068 | - | 0,303 | **0,033** | **0,035** |
| Putamen_L | 0,215 | 0,444 | **0,025** | 0,168 | 0,156 |
| Putamen_R | 0,256 | 0,567 | **0,002** | 0,387 | 0,359 |
| Rolandic_Oper_L | 0,338 | - | **0,003** | 0,125 | 0,126 |
| Rolandic_Oper_R | **0,013** | - | 0,754 | **0,014** | **0,015** |
| Supp_Motor_Area_L | **0,008** | **0,047** | **0,016** | **0,006** | **0,006** |
| Supra_Marginal_L | 0,337 | - | **0,025** | 0,147 | 0,150 |
| Supra_Marginal_R | **0,000** | 0,714 | **0,000** | **0,000** | **0,000** |
| Temporal_Inf_L | 0,097 | - | 0,147 | **0,027** | **0,028** |
| Temporal_Inf_R | 0,140 | - | **0,000** | 0,878 | 0,886 |
| Temporal_Mid_R | **0,019** | - | 0,447 | **0,034** | **0,036** |
| Temporal_Pole_Mid_L | 0,195 | - | **0,047** | 0,185 | 0,184 |
| Temporal_Pole_Mid_R | **0,000** | 0,826 | **0,050** | **0,001** | **0,001** |
| Temporal_Pole_Sup_L | 0,250 | - | **0,002** | 0,232 | 0,229 |
| Temporal_Pole_Sup_R | **0,000** | - | **0,000** | **0,000** | **0,000** |

**Table S4.** R squared and p-values for the independent variable age for the regions that had at least one significant (ANCOVA, p<0.05) network measure (in bold p-values smaller than 0.05)

|  | STR | | BC | | EC | | CLU | | EFI | |
| --- | --- | --- | --- | --- | --- | --- | --- | --- | --- | --- |
| AAL Region | R Squared | Sig. | R Squared | Sig. | R Squared | Sig. | R Squared | Sig. | R Squared | Sig. |
| Calcarine_R | 0,107 | 0,302 | 0,054 | 0,999 | 0,227 | **0,000** | 0,100 | 0,326 | 0,099 | 0,334 |
| Caudate_L | 0,181 | **0,038** | 0,082 | 0,574 | 0,220 | **0,000** | 0,187 | **0,042** | 0,182 | **0,049** |
| Caudate_R | 0,291 | **0,000** | - | - | 0,320 | **0,000** | 0,294 | **0,000** | 0,289 | **0,001** |
| Cerebellum_6_L | 0,131 | **0,019** | - | - | 0,160 | **0,013** | 0,128 | **0,034** | 0,126 | **0,039** |
| Cerebellum_8_L | 0,109 | 0,444 | - | - | 0,257 | **0,041** | 0,092 | 0,617 | 0,093 | 0,613 |
| Cerebellum_8_R | 0,087 | 0,566 | - | - | 0,181 | **0,002** | 0,083 | 0,669 | 0,083 | 0,674 |
| Cerebellum_Crus2_L | 0,119 | 0,646 | - | - | 0,148 | **0,020** | 0,121 | 0,660 | 0,118 | 0,675 |
| Cingulum_Ant_L | 0,226 | **0,001** | - | - | 0,358 | **0,000** | 0,196 | **0,011** | 0,189 | **0,015** |
| Cingulum_Ant_R | 0,111 | 0,274 | - | - | 0,141 | **0,012** | 0,109 | 0,414 | 0,106 | 0,432 |
| Cingulum_Mid_L | 0,120 | **0,045** | - | - | 0,140 | 0,064 | 0,115 | 0,121 | 0,113 | 0,133 |
| Cingulum_Mid_R | 0,129 | **0,040** | - | - | 0,182 | **0,026** | 0,121 | 0,078 | 0,119 | 0,086 |
| Cuneus_R | 0,140 | **0,019** | 0,462 | **0,000** | 0,179 | **0,001** | 0,128 | 0,060 | 0,125 | 0,066 |
| Frontal_Inf_Oper_L | 0,114 | 0,134 | 0,238 | **0,000** | 0,137 | **0,015** | 0,110 | 0,164 | 0,110 | 0,169 |
| Frontal_Inf_Orb_L | 0,095 | 0,457 | - | - | 0,150 | **0,045** | 0,100 | 0,491 | 0,098 | 0,504 |
| Frontal_Inf_Orb_R | 0,109 | 0,366 | - | - | 0,137 | **0,012** | 0,112 | 0,445 | 0,109 | 0,455 |
| Frontal_Inf_Tri_L | 0,109 | 0,082 | 0,127 | **0,020** | 0,123 | **0,040** | 0,106 | 0,144 | 0,105 | 0,155 |
| Frontal_Inf_Tri_R | 0,263 | **0,003** | 0,110 | 0,952 | 0,312 | **0,000** | 0,241 | **0,031** | 0,236 | **0,037** |
| Frontal_Mid_L | 0,120 | 0,206 | - | - | 0,139 | **0,010** | 0,122 | 0,267 | 0,120 | 0,281 |
| Frontal_Mid_R | 0,119 | 0,441 | - | - | 0,156 | **0,036** | 0,125 | 0,389 | 0,122 | 0,402 |
| Frontal_Sup_L | 0,129 | **0,017** | 0,091 | 0,580 | 0,135 | **0,005** | 0,131 | **0,043** | 0,130 | **0,045** |
| Frontal_Sup_Medial_L | 0,143 | 0,100 | 0,066 | 0,957 | 0,177 | **0,003** | 0,135 | 0,143 | 0,133 | 0,149 |
| Frontal_Sup_Medial_R | 0,104 | 0,210 | - | - | 0,180 | **0,000** | 0,100 | 0,299 | 0,099 | 0,306 |
| Frontal_Sup_Orb_L | 0,135 | 0,123 | 0,037 | 1,000 | 0,134 | **0,037** | 0,131 | 0,203 | 0,129 | 0,213 |
| Hippocampus_L | 0,136 | 0,165 | - | - | 0,186 | **0,023** | 0,133 | 0,218 | 0,130 | 0,226 |
| Hippocampus_R | 0,150 | **0,050** | 0,503 | **0,000** | 0,258 | **0,000** | 0,139 | 0,055 | 0,136 | 0,060 |
| Insula_L | 0,250 | **0,000** | - | - | 0,383 | **0,000** | 0,236 | **0,000** | 0,228 | **0,000** |
| Insula_R | 0,266 | **0,000** | - | - | 0,442 | **0,000** | 0,245 | **0,000** | 0,237 | **0,000** |
| Occipital_Sup_L | 0,149 | **0,001** | 0,299 | **0,000** | 0,167 | **0,000** | 0,141 | **0,010** | 0,138 | **0,012** |
| Occipital_Sup_R | 0,151 | **0,005** | 0,046 | 1,000 | 0,198 | **0,000** | 0,143 | **0,030** | 0,140 | **0,035** |
| ParaHippocampal_L | 0,127 | **0,028** | - | - | 0,148 | **0,000** | 0,124 | 0,071 | 0,122 | 0,082 |
| ParaHippocampal_R | 0,121 | 0,053 | - | - | 0,124 | **0,031** | 0,121 | 0,087 | 0,119 | 0,096 |
| Parietal_Inf_L | 0,089 | 0,750 | 0,130 | **0,028** | 0,133 | 0,257 | 0,084 | 0,823 | 0,083 | 0,825 |
| Precentral_L | 0,125 | 0,257 | 0,071 | 0,898 | 0,171 | **0,002** | 0,119 | 0,428 | 0,117 | 0,433 |
| Precentral_R | 0,134 | 0,106 | 0,145 | **0,002** | 0,139 | 0,338 | 0,133 | 0,153 | 0,132 | 0,154 |
| Precuneus_R | 0,100 | 0,217 | - | - | 0,116 | **0,050** | 0,099 | 0,350 | 0,098 | 0,359 |
| Putamen_R | 0,143 | 0,304 | 0,175 | 0,060 | 0,271 | **0,001** | 0,144 | 0,152 | 0,145 | 0,154 |
| Supp_Motor_Area_L | 0,133 | 0,102 | 0,093 | 0,449 | 0,157 | **0,001** | 0,128 | 0,187 | 0,126 | 0,195 |
| Supp_Motor_Area_R | 0,093 | 0,424 | 0,100 | 0,145 | 0,117 | **0,031** | 0,094 | 0,509 | 0,093 | 0,517 |
| Supra_Marginal_R | 0,126 | 0,066 | 0,502 | **0,000** | 0,161 | 0,151 | 0,117 | 0,159 | 0,115 | 0,169 |
| Temporal_Pole_Mid_L | 0,111 | 0,183 | - | - | 0,148 | **0,001** | 0,106 | 0,272 | 0,104 | 0,294 |
| Temporal_Pole_Mid_R | 0,134 | 0,468 | 0,076 | 0,740 | 0,167 | **0,001** | 0,121 | 0,620 | 0,119 | 0,639 |
| Temporal_Sup_L | 0,104 | 0,494 | 0,331 | **0,000** | 0,208 | **0,036** | 0,093 | 0,544 | 0,093 | 0,543 |
| Thalamus_L | 0,289 | **0,003** | 0,117 | 0,688 | 0,359 | **0,003** | 0,320 | **0,005** | 0,313 | **0,007** |
| Thalamus_R | 0,284 | **0,006** | 0,137 | 0,833 | 0,364 | **0,000** | 0,326 | **0,015** | 0,319 | **0,019** |
